# Galvanic Vestibular-Evoked Myogenic Potentials in Unilateral Vestibular Schwannoma: Paired-Ear Differences and Contralateral Abnormalities

**DOI:** 10.3390/diagnostics16213663

**Published:** 2026-11-05

**Authors:** Chih-Ming Chang, Wu-Chia Lo, Yi-Ho Young, Li-Jen Liao, Ping-Chia Cheng, Po-Wen Cheng

**Affiliations:** 1 Department of Biomedical Engineering, National Yang Ming Chiao Tung University, Taipei 112304, Taiwan; b88401077@ntu.edu.tw (C.-M.C.); i.cruising@gmail.com (P.-C.C.); 2 Department of Otolaryngology, Far Eastern Memorial Hospital, Taipei 220216, Taiwan; lowuchia@gmail.com (W.-C.L.); youngyh@ntu.edu.tw (Y.-H.Y.); deniro@mail2000.com.tw (L.-J.L.); 3 Department of Otolaryngology, National Taiwan University Hospital, Taipei 100225, Taiwan; 4 Graduate Institute of Medicine, Yuan Ze University, Taoyuan 320315, Taiwan; 5 Department of Electrical Engineering, Yuan Ze University, Taoyuan 320315, Taiwan

**Keywords:** galvanic vestibular stimulation, cervical vestibular-evoked myogenic potential, ocular vestibular-evoked myogenic potential, vestibular schwannoma, aging

## Abstract

**Background/Objectives:** To characterize paired-ear vestibular-evoked myogenic potential (VEMP) response patterns and contralateral abnormalities in patients with MRI-confirmed unilateral vestibular schwannoma (VS). **Methods:** This exploratory retrospective paired-ear study included 24 patients with unilateral VS confirmed by contrast-enhanced MRI. Patients underwent pretreatment pure-tone audiometry, caloric testing, air-conducted sound cervical VEMP (ACS-cVEMP), bone-conducted vibration ocular VEMP (BCV-oVEMP), galvanic vestibular stimulation cervical VEMP (GVS-cVEMP), and GVS-oVEMP. Tumor-affected and contralateral non-tumor ears were compared using McNemar’s and Wilcoxon signed-rank tests. Age was compared by contralateral VEMP status using Mann–Whitney U tests. All *p* values were unadjusted for multiple comparisons. **Results**: Hearing loss and caloric abnormalities were more frequent in tumor-affected ears (91.7% vs. 20.8% and 62.5% vs. 8.3%, respectively; both *p* < 0.001). Abnormality rates were higher in tumor-affected than contralateral non-tumor ears for ACS-cVEMP (83.3% vs. 45.8%; *p* = 0.022), GVS-cVEMP (70.8% vs. 20.8%; *p* = 0.002), and GVS-oVEMP (70.8% vs. 16.7%; *p* = 0.002), whereas the BCV-oVEMP difference was not significant (62.5% vs. 33.3%; *p* = 0.092). Tumor-affected ears showed prolonged GVS-cVEMP p13 latency (median, 12.47 vs. 11.35 ms; *p* = 0.008) and reduced amplitude (median, 59.02 vs. 66.24 μV; *p* = 0.044). Patients with contralateral ACS-cVEMP abnormalities were older than those with normal responses (median, 62 vs. 45 years; *p* = 0.001); age did not differ significantly by contralateral GVS-VEMP status. **Conclusions:** In this exploratory paired-ear study, GVS-cVEMP abnormalities were more frequent in tumor-affected ears, and the observed contralateral abnormality rate was numerically lower than that for ACS-cVEMP. GVS-cVEMP may complement conventional VEMPs when contralateral abnormalities complicate interpretation. These preliminary findings require validation in larger prospective controlled studies.

## 1. Introduction

Vestibular schwannoma (VS) is a benign and typically slow-growing intracranial tumor arising from Schwann cells along either the superior or inferior vestibular nerve and accounts for approximately 8–10% of adult intracranial tumors [1]. Although contrast-enhanced magnetic resonance imaging remains the diagnostic gold standard [2], audiovestibular tests provide complementary information regarding cochleovestibular function.

Alternative screening tests, including auditory brainstem response (ABR) [3,4] and caloric testing [1,5], have been used to evaluate cochlear nerve and superior vestibular nerve involvement, respectively. More recently, vestibular-evoked myogenic potentials (VEMPs), including cervical VEMP (cVEMP) [6] and ocular VEMP (oVEMP) [7], have been incorporated into vestibular test batteries to assess otolith-related reflex pathways in VS patients [8,9]. cVEMPs recorded from the ipsilateral sternocleidomastoid (SCM) muscle predominantly reflect the uncrossed sacculo-collic reflex (SCR) pathway [10], whereas oVEMPs recorded from the contralateral infraocular muscles mainly represent the crossed vestibulo-ocular reflex (VOR) pathway [11]. Conventional VEMPs elicited by air-conducted sound (ACS) or bone-conducted vibration (BCV) may not reliably distinguish otolithic end-organ dysfunction from retrolabyrinthine neural involvement. This distinction is particularly important in VS because the tumor is neural in origin. Galvanic vestibular stimulation (GVS) VEMPs that bypass sound transmission and activate vestibular afferents may provide complementary information about vestibular afferent pathways. GVS-evoked cVEMPs and oVEMPs have been applied in various audiovestibular disorders to assess residual vestibular nerve function and to differentiate receptor-level dysfunction from retrolabyrinthine impairment [12,13,14,15,16,17,18]. In unilateral VS, comparing conventional and GVS-VEMP responses may help characterize vestibular pathway function on each side in relation to the MRI-defined tumor side. A recent study evaluated GVS-VEMPs for predicting the nerve of tumor origin and residual vestibular nerve function in patients with VS. However, paired-ear contralateral abnormality patterns and their potential association with age were not its primary focus [19].

VEMP responses are known to decline with aging, including reduced response rates, prolonged latencies, and decreased amplitudes [20,21,22]. In older patients with unilateral VS, contralateral VEMP abnormalities may not necessarily indicate bilateral tumor-related dysfunction; instead, they may reflect aging or other non-lesional factors. Despite the potential advantages of galvanic stimulation, the clinical role of GVS-VEMPs in VS patients has not been fully established. On this basis, the primary objective of this study was to characterize paired-ear VEMP response patterns and contralateral abnormalities in patients with MRI-confirmed unilateral VS. Secondary exploratory analyses assessed associations between age and contralateral abnormality status.

## 2. Materials and Methods

### 2.1. Patients

This retrospective study was conducted at a single tertiary referral center and included 24 patients with unilateral VS confirmed by gadolinium-enhanced MRI. Patients with concomitant ear diseases, bilateral vestibular lesions, or central nervous system disorders were excluded. All patients underwent a battery of inner ear function tests, including pure-tone audiometry (PTA), ACS-cVEMP, BCV-oVEMP, GVS-cVEMP, GVS-oVEMP, and bithermal caloric testing before treatment. Ears were classified as tumor-affected or contralateral non-tumor ears according to MRI findings. The analyses described functional differences between these sides; VEMP-based prediction of tumor side or the anatomical nerve of origin was not evaluated. The study protocol was conducted in accordance with the Declaration of Helsinki and approved by the Research Ethics Review Committee at Far Eastern Memorial Hospital (No. 109143-E). The requirement for informed consent was waived by the committee because of the retrospective design and use of de-identified data.

### 2.2. PTA

PTA was performed to determine the mean hearing level. The pure-tone average was calculated as the mean hearing threshold at frequencies of 0.5, 1, 2, and 3 kHz. Hearing loss was defined as a mean hearing level greater than 25 dB HL.

### 2.3. Bithermal Caloric Testing

Bithermal caloric testing was conducted with patients lying in the supine position. The external auditory canal was irrigated with cold water at 30 °C and warm water at 44 °C. During the examination, the head was elevated 30° above the horizontal plane to optimize stimulation of the horizontal semicircular canal. Induced eye movements were recorded using videonystagmography.

Canal paresis was determined when the maximal interaural difference in slow-phase velocity exceeded 25% of the sum of bilateral slow-phase velocities. In cases where no measurable response was elicited by conventional bithermal caloric irrigation, ice-water irrigation of the external auditory canal was performed to confirm caloric areflexia.

### 2.4. ACS-cVEMP Testing

ACS-cVEMP responses were recorded from the SCM muscle ipsilateral to the stimulated site. A surface recording electrode was attached to the upper half of the SCM muscle, while the reference electrode was positioned over the lateral end of the upper sternum. The ground electrode was placed at the suprasternal notch. SCM electromyographic (EMG) activity was collected, filtered using a band-pass setting of 20 to 2000 Hz, and amplified.

Acoustic stimulation was delivered monaurally to the tested ear using click stimuli at an intensity of 105 dB nHL, a stimulation rate of 5 Hz, and a duration of 0.5 ms. During data acquisition, participants lay in the supine position and were asked to lift their heads in order to maintain adequate tonic contraction of the SCM muscle. For each recording, 128 responses were averaged, and the final ACS-cVEMP waveform was obtained by averaging two reproducible trials.

### 2.5. BCV-oVEMP Testing

BCV-oVEMP responses were recorded beneath each eye during stimulation at the midline Fpz site. Responses recorded beneath the right and left eyes were assigned to the left and right vestibular sides, respectively. The active electrode was placed immediately beneath the recording eye, with the reference electrode positioned 1–2 cm below it. The ground electrode was placed at the suprasternal notch. EMG signals were recorded, band-pass filtered between 1 and 1000 Hz, and amplified.

BCV was applied using a hand-held electromechanical vibrator. The stimulus was delivered at a repetition rate of 5 Hz with a duration of 0.5 ms. The input signal was generated through a customized amplifier system, and the driving voltage was adjusted to achieve a peak force of 12 N. During stimulation, most of the vibrator’s weight was supported by the examiner, and the bakelite cap attached to the vibrator was kept perpendicular to the skull surface. Throughout the recording, subjects were instructed to fix their gaze upward on a small target positioned more than 2 m away, corresponding to a vertical gaze angle of approximately 30–35 degrees above the horizontal plane. Each run consisted of the averaged response to 50 stimuli, and two repeatable runs were averaged to generate the final BCV-oVEMP waveform.

### 2.6. GVS-cVEMP Testing

For GVS-cVEMP testing, the electrode montage and EMG recording settings were the same as those used for ACS-cVEMP recording. Galvanic stimulation was delivered through electrodes placed over the forehead and the mastoid process of the tested side, serving as the anode and cathode, respectively.

Recordings were performed with subjects in the supine position. Galvanic stimuli were presented at an intensity of 5 mA, a stimulation rate of 5 Hz, and a duration of 1 ms. Data were acquired under two conditions: with active SCM contraction and without SCM contraction. To reduce contamination from electrical artifacts in the GVS-cVEMP recordings, the waveform obtained in the absence of SCM contraction was subtracted from that recorded during SCM contraction. Responses to 128 galvanic stimuli were averaged for each run, and two reproducible runs were combined to obtain the final GVS-cVEMP waveform.

### 2.7. GVS-oVEMP Testing

For GVS-oVEMP recording, the electrode placement and EMG acquisition parameters were identical to those described for BCV-oVEMP testing. The galvanic stimulation electrodes were positioned with the anode on the forehead and the cathode on the mastoid process of the tested side.

Subjects were tested in the sitting position. Galvanic stimulation was delivered at 5 mA with a repetition rate of 5 Hz and a stimulus duration of 1 ms. Recordings were obtained during both upward and downward gaze. To minimize electrical artifacts, the responses recorded during downward gaze were subtracted from those obtained during upward gaze. For each run, responses to 128 galvanic stimuli were averaged, and the final GVS-oVEMP waveform was derived from the average of two reproducible runs. For both GVS-cVEMP and GVS-oVEMP, repeated recordings were used to assess waveform repeatability within a single examination; formal quantitative test–retest reliability was not evaluated.

### 2.8. Assessment of VEMPs

**Figure 1:**
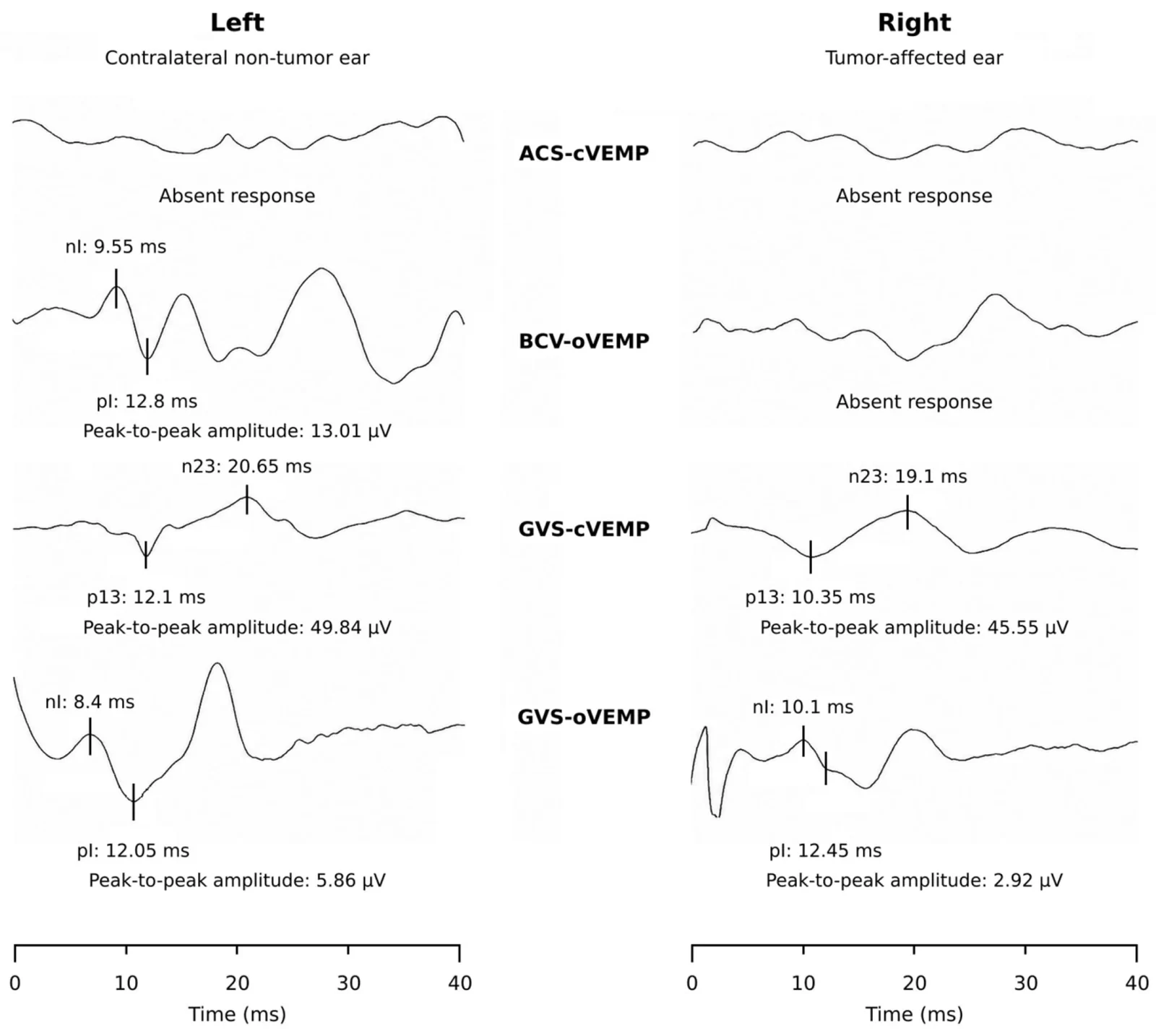
Representative VEMP recordings from a patient with a right vestibular schwannoma measuring 1.4 × 0.5 cm. The left and right columns show the contralateral non-tumor side and tumor-affected side, respectively. For cVEMPs, recordings were obtained from the SCM ipsilateral to the assessed vestibular side. For oVEMPs, responses recorded beneath the right and left eyes were assigned to the left and right vestibular sides, respectively. ACS-cVEMP responses are absent bilaterally, and the right BCV-oVEMP response is absent. GVS-cVEMP responses are present bilaterally. The right GVS-oVEMP peak-to-peak amplitude (nI-pI amplitude) is reduced relative to the left (2.92 versus 5.86 μV). Peak labels identify p13 and n23 for cVEMPs and nI and pI for oVEMPs. Latencies are expressed in ms and peak-to-peak amplitudes in μV. ACS, air-conducted sound; BCV, bone-conducted vibration; GVS, galvanic vestibular stimulation; cVEMP, cervical vestibular-evoked myogenic potential; oVEMP, ocular vestibular-evoked myogenic potential.

Responses were interpreted using previously established laboratory reference values [12,13,14,15,16]. Prolonged latency was defined as p13 >14.6 ms for ACS-cVEMP, p13 >13.2 ms for GVS-cVEMP, nI >12.6 ms for BCV-oVEMP, or nI >10.4 ms for GVS-oVEMP. The interaural amplitude-asymmetry ratio was the absolute bilateral amplitude difference divided by the summed bilateral amplitude. For bilaterally recordable responses, an amplitude asymmetry ratio >0.33 was classified as an amplitude abnormality in the ear with the lower peak-to-peak amplitude. Non-recordable responses were categorized as absent and were reported separately from latency and amplitude-asymmetry abnormalities. A VEMP result was classified as abnormal when latency was delayed, amplitude asymmetry was abnormal, or the response was absent (Figure 1).

### 2.9. Statistical Analysis

Statistical analyses were performed using IBM SPSS Statistics 23. For categorical analyses, absent responses were classified as abnormal. McNemar’s test was used for paired comparisons of abnormality rates between tumor-affected and contralateral non-tumor ears. Paired effect estimates were summarized as absolute differences in abnormality rates and matched odds ratios. The matched odds ratio was defined as the number of patients with an abnormal response only in the tumor-affected ear divided by the number with an abnormal response only in the contralateral non-tumor ear. Exact conditional 95% confidence intervals for the matched odds ratios were calculated from the discordant paired counts. Confidence intervals were not adjusted for multiple comparisons. For quantitative paired-ear analyses, latency and amplitude were analyzed by the Wilcoxon signed-rank test only when responses were recordable in both ears; non-recordable responses were not assigned quantitative values and were excluded from the corresponding paired analysis. The number of analyzable paired observations varied across VEMP parameters because non-recordable responses were excluded from quantitative analyses. To evaluate the association between age and contralateral VEMP abnormalities, age distributions were compared between patients with and without contralateral abnormalities using the Mann–Whitney U test.

All *p* values were two-sided and unadjusted for multiple comparisons. The categorical, quantitative, and age analyses were considered exploratory. No formal multiplicity adjustment was applied; therefore, the risk of false-positive findings across the comparisons was not formally controlled. Findings meeting *p* < 0.05 were regarded as nominally significant and hypothesis-generating rather than confirmatory. Related nominal findings were not treated as independent evidence of comparative diagnostic superiority. Reported confidence intervals were also unadjusted for multiplicity.

### 2.10. AI Disclosure Statement

During the preparation of this manuscript, the authors used ChatGPT (OpenAI) only for language editing and improving clarity. The authors reviewed and edited the manuscript before submission and retain full responsibility for its scientific content, accuracy, interpretation, and final wording.

## 3. Results

A total of 24 patients with unilateral VS were included in the final analysis (Table 1). The cohort comprised 16 women and 8 men, with a mean age of 52.3 ± 13.8 years. The mean tumor size was 2.11 ± 1.51 cm, with a median of 2.0 cm.

Abnormal audiovestibular test results were more frequent in the tumor-affected ears than in the contralateral non-tumor ears (Table 2). On the tumor-affected side, abnormality rates were 91.7% for PTA, 62.5% for caloric testing, 83.3% for ACS-cVEMP, 62.5% for BCV-oVEMP, 70.8% for GVS-cVEMP, and 70.8% for GVS-oVEMP. The corresponding abnormality rates on the contralateral non-tumor ears were 20.8%, 8.3%, 45.8%, 33.3%, 20.8%, and 16.7%, respectively. In unadjusted analyses, paired-ear differences were observed for hearing loss (*p* < 0.001), caloric abnormality (*p* < 0.001), ACS-cVEMP abnormality (*p* = 0.022), GVS-cVEMP abnormality (*p* = 0.002), and GVS-oVEMP abnormality (*p* = 0.002). In contrast, the difference for BCV-oVEMP abnormality did not reach statistical significance (*p* = 0.092).

The absolute paired differences in abnormality rates, calculated as tumor-affected minus contralateral non-tumor ear rates, were 37.5, 29.2, 50.0, and 54.2 percentage points for ACS-cVEMP, BCV-oVEMP, GVS-cVEMP, and GVS-oVEMP, respectively. The corresponding matched odds ratios were 5.50 (exact 95% CI, 1.20–51.07), 3.33 (0.86–18.85), 13.00 (1.95–552.47), and 7.50 (1.74–67.59), respectively. These within-modality estimates do not directly compare diagnostic performance between modalities.

Among patients with bilaterally recordable responses for each modality, quantitative paired-ear comparisons (Table 3) further showed that ACS-cVEMP amplitude was lower in the tumor-affected ears than the contralateral non-tumor ears (median, 57.10 vs. 171.75 μV; *p* = 0.008). For BCV-oVEMP, the tumor-affected ears demonstrated a prolonged nI latency (median, 9.14 vs. 8.80 ms; *p* = 0.047). For GVS-cVEMP, the tumor-affected ears showed both prolonged p13 latency (median, 12.47 vs. 11.35 ms; *p* = 0.008) and reduced amplitude (median, 59.02 vs. 66.24 μV; *p* = 0.044). No statistically significant between-side differences were identified in the quantitative parameters of GVS-oVEMP. Among the 20 tumor-affected ears with abnormal ACS-cVEMP responses, 16 (80%) also had abnormal GVS-cVEMP responses, whereas 4 (20%) had preserved GVS-cVEMP responses.

**Table 3:** Quantitative paired-ear vestibular-evoked myogenic potential comparisons.

Parameter	Paired Observations, *n*	Tumor-Affected Ears, Median	Contralateral Non-Tumor Ears, Median	*p* Value
**ACS-cVEMP p13 Latency (ms)**	9	11.95	11.20	0.066
**ACS-cVEMP Amplitude (μV)**	9	57.10	171.75	0.008
**BCV-oVEMP nI Latency (ms)**	10	9.14	8.80	0.047
**BCV-oVEMP Amplitude (μV)**	10	5.20	3.20	0.126
**GVS-cVEMP p13 Latency (ms)**	20	12.47	11.35	0.008
**GVS-cVEMP Amplitude (μV)**	20	59.02	66.24	0.044
**GVS-oVEMP nI Latency (ms)**	9	8.30	8.50	0.139
**GVS-oVEMP Amplitude (μV)**	9	2.60	2.48	0.214

*p* values were calculated using the Wilcoxon signed-rank test. ACS: air-conducted sound, BCV: bone-conducted vibration, GVS: galvanic vestibular stimulation. cVEMP: cervical vestibular-evoked myogenic potential. oVEMP: ocular vestibular-evoked myogenic potential.

In unadjusted analyses, patients with abnormal ACS-cVEMPs in the contralateral non-tumor ear were older than those with normal contralateral ACS-cVEMPs (n = 11 vs. 13, median age = 62 vs. 45 years, *p* = 0.001). In contrast, median age did not differ significantly between patients with abnormal and normal contralateral responses for BCV-oVEMP (n = 8 vs. 16, median age = 54 vs. 56 years, *p* = 0.697), GVS-cVEMP (n = 5 vs. 19, median age = 55 vs. 54 years, *p* = 0.679), or GVS-oVEMP (n = 4 vs. 20, median age = 52.5 vs. 56.5 years, *p* = 0.431).

## 4. Discussion

This paired-ear analysis revealed differences between tumor-affected and contralateral non-tumor ears in abnormality rates for PTA, caloric testing, ACS-cVEMP, GVS-cVEMP, and GVS-oVEMP, supporting the presence of predominantly side-specific audiovestibular dysfunction across both auditory and vestibular pathways in unilateral VS. More importantly, these findings highlight that lateralization of vestibular pathway dysfunction may be complicated by abnormalities detected in the contralateral non-tumor ear. However, the detection of contralateral abnormalities does not establish a causal effect of the unilateral tumor on the opposite vestibular system.

Among the VEMP modalities, GVS-cVEMP was the only modality to show nominally significant differences between tumor-affected and contralateral non-tumor ears in abnormality rate, p13 latency, and amplitude in unadjusted analyses. GVS-cVEMP showed a numerically lower contralateral abnormality rate than ACS-cVEMP. The present data do not establish the reasons for this difference or whether it reflects different age effects, stimulus characteristics, or recording conditions.

The oVEMP results were less straightforward. BCV-oVEMP showed prolonged nI latency in tumor-affected ears but no statistically significant difference in abnormality rate, whereas GVS-oVEMP showed a categorical difference without statistically significant quantitative between-side differences. Therefore, oVEMP responses may still contribute useful physiological information. The categorical and quantitative response patterns differed across modalities, but their comparative diagnostic performance was not formally evaluated. The discrepancy between categorical and quantitative findings may reflect limited paired data after exclusion of absent responses, as well as variability in superior vestibular pathway involvement and oVEMP recording conditions.

Concurrent ACS-cVEMP and GVS-cVEMP abnormalities were frequently observed in tumor-affected ears, whereas abnormal ACS-cVEMP with preserved GVS-cVEMP was uncommon. This pattern is compatible with differential physiological involvement beyond the otolithic receptor level [18]. However, without surgical or histopathological confirmation, the findings cannot establish the anatomical site of dysfunction or the nerve of tumor origin. Clinically, GVS-cVEMP may complement conventional VEMP testing when contralateral abnormalities complicate interpretation in unilateral VS, but its diagnostic role remains exploratory.

Patients with contralateral ACS-cVEMP abnormalities were older than those with normal contralateral responses in unadjusted analyses, whereas no statistically significant differences in age were observed between patients with and without contralateral GVS-cVEMP or GVS-oVEMP abnormalities. Given the small subgroup sizes and absence of independent age-matched healthy controls, the observed association between age and contralateral ACS-cVEMP abnormality status should be regarded as exploratory. The non-significant age comparisons for GVS-cVEMP and GVS-oVEMP do not establish that these responses are unaffected by aging or less susceptible to age-related changes than ACS-cVEMP. Differences in statistical significance across modalities do not demonstrate differences in their age effects. Larger studies with age-matched healthy controls and direct comparisons of age-related effects across VEMP modalities are needed to clarify these relationships.

The present paired-ear study examined the potential role of GVS-VEMPs in lateralization of vestibular pathway dysfunction in unilateral VS. Previous studies have demonstrated abnormal cVEMP and/or oVEMP responses in patients with VS [8,9], and VEMP abnormalities may occasionally be the only detectable abnormality despite normal hearing and caloric findings [8]. However, these investigations primarily focused on identifying vestibular dysfunction rather than on the potential influence of contralateral abnormalities on lateralization of vestibular pathway dysfunction. GVS-cVEMP showed a numerically lower contralateral abnormality rate than ACS-cVEMP in this cohort. This observation describes a difference in the distribution of abnormal responses but does not establish superior diagnostic accuracy or more reliable assignment of tumor side. Differences in nominal statistical significance across modalities likewise do not demonstrate differences in diagnostic performance. Whether adding GVS-cVEMP improves assessment beyond conventional VEMP testing should be investigated in prospective studies using predefined rules for assigning tumor side and direct paired comparisons of classification performance. Its contribution to clinical decision-making and patient outcomes also remains to be established.

The numerically higher abnormality rate of ACS-cVEMP than of BCV-oVEMP in the tumor-affected ears was noted; however, this finding should be interpreted with caution and should not be regarded as direct evidence of anatomical tumor origin. Earlier studies have indicated that the reliability of VEMP-based prediction of the anatomical nerve of origin remains controversial [1]. Operative identification of the nerve of tumor origin was unavailable in the present cohort. The electrophysiological findings should therefore be interpreted as functional response patterns relative to the MRI-defined tumor side, rather than validated evidence of superior or inferior vestibular nerve origin.

Several clinical implications can be drawn. First, GVS-cVEMP may complement conventional VEMP testing when contralateral abnormalities complicate interpretation in unilateral VS. Its lower observed rate of contralateral abnormalities may aid interpretation. However, its diagnostic value remains exploratory and requires confirmation in larger prospective studies with age-matched controls and surgical correlation. Second, the discrepancy between categorical and quantitative oVEMP findings suggests that different VEMP modalities may contribute differently depending on whether the clinical aim is to screen for abnormal responses, assess lateralization of vestibular pathway dysfunction, or obtain a more detailed physiological profile of vestibular nerve dysfunction. Finally, concurrent ACS-cVEMP and GVS-cVEMP abnormalities are compatible with dysfunction of the tested vestibular reflex pathways, but do not establish an exclusively retrolabyrinthine mechanism or identify the anatomical nerve of tumor origin.

This study was limited by its small retrospective single-center design, which may have introduced selection bias and limited statistical power. Referral patterns and the availability of complete pretreatment audiovestibular data may have influenced patient inclusion, and the resulting cohort may not represent the broader population of patients with VS. The small sample limits estimation precision and statistical power, particularly for quantitative analyses restricted to patients with bilaterally recordable responses and for subgroup comparisons. The wide confidence intervals indicate substantial uncertainty in the magnitude of the observed associations. Non-significant findings should not be interpreted as demonstrating equivalence or the absence of an effect. Multiple comparisons were performed without formal adjustment for multiplicity, increasing the risk of false-positive findings. Some nominally significant results may not remain statistically significant after adjustment. Accordingly, both nominally significant and non-significant results require cautious interpretation. Findings across related VEMP parameters should not be interpreted as independent confirmation of superiority, and the present results require independent validation. The absence of independent age-matched healthy controls limits assessment of whether contralateral abnormalities exceeded those expected for age. The present data therefore cannot establish age independence or reduced susceptibility to aging for GVS-VEMPs. Formal diagnostic accuracy and incremental clinical value were not assessed. The observed abnormality rates and matched odds ratios should therefore be interpreted as descriptions of paired-ear functional asymmetry rather than evidence of superior diagnostic performance. Operative identification of the nerve of tumor origin was unavailable; therefore, prediction of superior or inferior vestibular nerve origin could not be validated. The paired-ear comparisons describe functional differences relative to the known MRI tumor side and do not establish the precise anatomical site of dysfunction. Quantitative analyses were restricted to patients with bilaterally recordable responses and therefore describe selected subsets of the cohort. Because non-recordability may be associated with underlying dysfunction or recording conditions, this restriction may introduce selection bias and alter the estimated between-ear differences. The direction and magnitude of this potential bias cannot be determined from the present analyses. Although absent responses were retained as abnormal outcomes in the categorical analyses, this does not eliminate selection bias from the quantitative estimates. Accordingly, quantitative findings should be interpreted together with the categorical analyses, in which absent responses were retained as abnormal outcomes. Formal quantitative test–retest reliability was not assessed. Although replicate runs were obtained within a single examination, these do not establish between-session agreement or the stability of individual latency, amplitude, and abnormality classifications. These findings should therefore be regarded as preliminary and hypothesis-generating. Larger prospective multicenter studies with clearly defined eligibility criteria and standardized testing protocols are needed to assess the reproducibility and generalizability of the observed paired-ear response patterns. Studies with imaging- and surgery-correlated analyses are needed to validate the lesion-lateralizing value of GVS-VEMPs.

## 5. Conclusions

In this small exploratory paired-ear study, GVS-cVEMP abnormalities were more frequent in tumor-affected ears than in contralateral non-tumor ears, and the observed contralateral abnormality rate was numerically lower than that for ACS-cVEMP. These findings provide a preliminary characterization of paired-ear functional response patterns in unilateral VS. Larger prospective controlled studies with age-matched healthy controls are needed to validate these observations.

## Figures and Tables

**Table 1:** Demographic, tumor, and audiovestibular test characteristics.

No. of Patients	24
Sex, female/male	16/8
Age, mean ± SD, years	52.3 ± 13.8
Tumor Side, right/left	11/13
Tumor Size, mean ± SD, cm	2.11 ± 1.51
Pure-tone Audiometry of Tumor-affected Ears, normal/abnormal	2/22
Caloric Testing of Tumor-affected Ears, normal/canal paresis/absent	9/6/9
ACS-cVEMP of Tumor-affected Ears, normal/delayed/decreased/delayed and decreased/absent	4/2/3/0/15
BCV-oVEMP of Tumor-affected Ears, normal/delayed/decreased/delayed and decreased/absent	9/1/2/0/12
GVS-cVEMP of Tumor-affected Ears, normal/delayed/decreased/delayed and decreased/absent	7/4/6/3/4
GVS-oVEMP of Tumor-affected Ears, normal/delayed/decreased/delayed and decreased/absent	7/1/3/0/13

ACS: air-conducted sound; BCV: bone-conducted vibration; GVS: galvanic vestibular stimulation. cVEMP: cervical vestibular-evoked myogenic potential; oVEMP: ocular vestibular-evoked myogenic potential.

**Table 2:** Comparisons of abnormality rates between tumor-affected and contralateral non-tumor ears in patients with vestibular schwannoma.

		Pure-Tone Audiometry			Caloric Testing		
		Contralateral Non-Tumor Ears			Contralateral Non-Tumor Ears		
		Normal	Abnormal	Total	Normal	Abnormal	Total
**Tumor-affected Ears**	Normal	1	1	2 (8.3%)	9	0	9 (37.5%)
	Abnormal	18	4	22 (91.7%)	13	2	15 (62.5%)
	Total	19 (79.2%)	5 (20.8%)		22 (91.7%)	2 (8.3%)	
	*p* Value	<0.001			< 0.001		
		**ACS-cVEMP Testing**			**BCV-oVEMP Testing**		
		**Contralateral Non-tumor Ears**			**Contralateral Non-tumor Ears**		
		**Normal**	**Abnormal**	**Total**	**Normal**	**Abnormal**	**Total**
**Tumor-affected Ears**	Normal	2	2	4 (16.7%)	6	3	9 (37.5%)
	Abnormal	11	9	20 (83.3%)	10	5	15 (62.5%)
	Total	13 (54.2%)	11 (45.8%)		16 (66.7%)	8 (33.3%)	
	*p* Value	0.022			0.092		
		**GVS-cVEMP Testing**			**GVS-oVEMP Testing**		
		**Contralateral Non-tumor Ears**			**Contralateral Non-tumor Ears**		
		**Normal**	**Abnormal**	**Total**	**Normal**	**Abnormal**	**Total**
**Tumor-affected Ears**	Normal	6	1	7 (29.2%)	5	2	7 (29.2%)
	Abnormal	13	4	17 (70.8%)	15	2	17 (70.8%)
	Total	19 (79.2%)	5 (20.8%)		20 (83.3%)	4 (16.7%)	
	*p* Value	0.002			0.002		

*p* values were calculated using McNemar’s test. ACS: air-conducted sound; BCV: bone-conducted vibration; GVS: galvanic vestibular stimulation. cVEMP: cervical vestibular-evoked myogenic potential; oVEMP: ocular vestibular-evoked myogenic potential.

## Data Availability

The de-identified data generated and analyzed in this study are available from the corresponding author upon reasonable request. The data are not publicly available because they contain individual-level clinical and audiovestibular information from a small cohort of patients with a relatively uncommon condition, which may carry a residual risk of participant re-identification. In addition, this retrospective study was conducted under a waiver of informed consent, and the ethics approval did not authorize unrestricted public dissemination of participant-level data. Requests for access will be considered subject to approval by the Research Ethics Review Committee of Far Eastern Memorial Hospital and applicable institutional data-protection requirements.

## Abbreviations

The following abbreviations are used in this manuscript:

VS	Vestibular schwannoma
ABR	Auditory brainstem response
VEMP	Vestibular-evoked myogenic potentials
cVEMP	Cervical vestibular-evoked myogenic potentials
oVEMP	Ocular vestibular-evoked myogenic potentials
SCM	Sternocleidomastoid
SCR	Sacculo-collic reflex
VOR	Vestibulo-ocular reflex
ACS	Air-conducted sound
BCV	Bone-conducted vibration
GVS	Galvanic vestibular stimulation
PTA	Pure-tone audiometry
EMG	Electromyography
